# Absorbable Powder Haemostat Use in Minimally Invasive Thoracic Surgery

**DOI:** 10.3390/jcm14010085

**Published:** 2024-12-27

**Authors:** Sara Ricciardi, Akshay Jatin Patel, Danilo Alunni Fegatelli, Sara Volpi, Federico Femia, Lea Petrella, Andrea Bille, Giuseppe Cardillo

**Affiliations:** 1 San Camillo Forlanini Hospital, Circonvallazione Gianicolense 87, 00186 Rome, Italy; sricciardi2@scamilloforlanini.rm.it; 2 Guy’s and St Thomas’ NHS Foundation Trust, London SE1 7EH, UK; a.patel.2@bham.ac.uk (A.J.P.); federico.femia@gstt.nhs.uk (F.F.); andrea.bille@gstt.nhs.uk (A.B.); 3 University of Birmingham, Birmingham B15 2TT, UK; 4 Department of Life Sciences, Health and Health Professions, Link Campus University, 00165 Rome, Italy; danilo.alunnifegatelli@uniroma1.it; 5 University of Sapienza, 00185 Rome, Italy; lea.petrella@uniroma1.it; 6 UniCamillus-Saint Camillus International, University of Health Sciences, 00131 Rome, Italy

**Keywords:** haemostatic agent, haemostatic powder, minimally invasive surgery, lung cancer, short-term outcomes

## Abstract

**Background**: Significant intraoperative and postoperative blood loss are rare but possibly life-threatening complications after lung resection surgery either during open or minimally invasive procedures. Microporous Polysaccharide Haemospheres (ARISTA™AH) have demonstrated time-efficient haemostasis, lower postoperative blood volumes and a lower blood transfusion requirement, without any identified adverse events across other specialities. The primary aim of our study was to evaluate the impact of ARISTA™AH on short-term postoperative outcomes in thoracic surgery. Our secondary aim was to compare ARISTA™AH with other commonly used haemostatic agents. **Methods**: We retrospectively reviewed a prospectively collected database of consecutive early-stage lung cancer patients surgically treated in two European centres (October 2020–December 2022). Exclusion criteria included open surgery, patients with coagulopathy/anticoagulant medication, major intraoperative bleeding, non-anatomical lung resection and age <18 years. The cohort was divided into five groups according to the haemostatic agent that was used. Propensity score matching was used to estimate the effect of ARISTA™AH on various intra- and postoperative parameters (continuous and binary outcome modelling). **Results**: A total of 482 patients (M/F:223/259; VATS 97/RATS 385) with a mean age of 68.9 (±10.6) years were analysed. In 253 cases, ARISTA™AH was intraoperatively used to control bleeding. This cohort of patients had a significant reduction in total drain volume by 135 mls (standard error 53.9; *p* = 0.012). The use of ARISTA™AH did reduce the average length of a hospital stay (−1.47 days) and the duration of chest drainage (−0.596 days), albeit not significantly. In the ARISTA™AH group, we observed no postoperative bleeding, no blood transfusion requirement, no 30-day mortality and no requirement for redo surgery. The use of ARISTA™AH significantly reduced the odds of postoperative complications, as well as the need for transfusion and redo surgery. **Conclusions**: Our data showed that Microporous Polysaccharide Haemospheres are a safe and effective haemostatic device. Their use has a positive effect on the short-term postoperative outcomes of patients surgically treated for early-stage lung cancer.

## 1. Introduction

Minimally invasive surgical (MIS) approaches have significantly improved thoracic surgical practice. Backed by randomized evidence, they now represent the gold-standard surgical approach in early-stage lung cancer treatment [1,2]. Notwithstanding the important advantages conferred by MIS and technical and technological improvements, the incidence of complications is still high (24–41%), with a major complication rate of 1.5% [3].

Among complications, increased haemorrhagic output or postoperative bleeding may increase the risk of re-operation. Moreover, retained effusion can also affect the length of chest drain and the length of stay [4].

Effective intraoperative haemostasis can help to reduce the risks of serious complications, to prevent infections and to reduce the hospitalization. For those reasons, blood loss management is important, and several haemostats have been developed to help surgeons minimize blood loss and the risk of bleeding [5,6].

Topical haemostatic agents are applied for bleeding control during surgery when conventional methods (e.g., compression, ligation, clipping and electrocautery) are impractical or insufficient. Several haemostats, such as synthetic glues, liquid fibrin sealants, powder, oxidized cellulose fleece and cotton gauze, are commonly used to help achieve haemostasis [7,8,9].

One of the most commonly used haemostatic agents is a local haemostatic bioabsorbable gauze, Surgicel^®^ (Ethicon, NJ, USA), that provides a scaffold on which platelets begin to adhere and aggregate. Few data have been reported on Surgicel’s use in thoracic surgery.

In 2021, a position paper was published on the use of haemostatic powder in several specialties, including general surgery, gynaecology, urology, breast and thoracic surgery, showing benefits and recommendations. Regarding thoracic surgery, the panel of experts endorsed the use of haemostatic powders for oozing during lymphadenectomy, in decortication of the pleura and in patients undergoing extracorporeal membrane oxygenation during lung transplantation [10].

According to the available literature, there have been very few controlled clinical studies or large retrospective cohort analyses to assess the efficacy and safety of topical haemostatic agents in thoracic surgery.

A steering group of multidisciplinary European surgeons formulated twelve recommendations on the use of haemostatic powder, showing its safety and efficacy [10].

Moreover, the Italian society of thoracic surgeons (SICT) drew up a Delphi consensus on air leak and intraoperative bleeding. The panel of experts reported that the routine intraoperative use of topical haemostats is indicated for modest bleeding/hematoma of the pulmonary artery and slow ooze; haemostasis of vascular sutures; haemostasis of particularly “sensitive” areas (e.g., close to the oesophagus/phrenic/recurrent nerves); parenchymal losses and bleeding from the parenchymal suture line; area of mediastinal lymphadenectomy; small bleeding during lymphadenectomy; after parietal pleurectomy or decortication [11].

Since there are many different operations and haemostats available, it is mandatory that the surgeons know each product in order to identify the optimal haemostatic agent for each procedure (Table 1).

The aim of our study was to evaluate the safety and efficacy of ARISTA^TM^ haemostatic polysaccharide haemospheres and to compare this specific product with other haemostatic agents that are currently used in thoracic surgery.

## 2. Materials and Methods

All consecutive patients who received elective minimally invasive surgery for early-stage non-small cell lung cancer (NSCLC) in two European centres [San Camillo Forlanini Hospital (Rome, Italy) and Guys Hospital (London, UK)] from October 2020 to December 2022 were included in this study.

Indications, preoperative examinations and criteria for performing minimally invasive surgery were in line with the standard set out by the NCCN guidelines.

The type of surgical approach (video-assisted thoracoscopic surgery—VATS—or robotic assisted thoracoscopic surgery—RATS) was chosen according to the surgeons’ preference. All robotic lobectomies were performed with a Da Vinci Xi platform, with a standard totally endoscopic technique, through four intercostal incisions, under CO_2_ insufflation (5–8 mmHg), and without utility incision. In 169 cases, an extra utility port (15 mm trocar) was used. The VATS lobectomies were performed with a bi-portal or tri-portal approach as the standard D’Amico or Copenhagen approaches [12,13].

The primary aim of our study was to evaluate the impact of ARISTA™AH on short-term postoperative outcomes in thoracic surgery, and the secondary aim was to compare ARISTA™AH with other commonly used haemostatic agents.

### 2.1. Data Collection

Data on patients intraoperatively treated with haemostats were prospectively recorded. Demographic, clinical, surgical and pathological data were then retrospectively analysed from the surgical registry. All patients were stratified according to the haemostatic agent used during surgery.

We excluded patients who underwent open surgery or who underwent wedge resection, patients with coagulopathy or anticoagulant medication and patients <18 years. Additional exclusion criteria comprised a history of allergic reactions after application of human fibrinogen, human thrombin or collagen of any origin. Patients who experienced major intraoperative bleeding due to vascular injury were also excluded.

The haemostatic agents used during surgery were as follows:

- ARISTA™ AH (BD, 1 Becton Drive, Franklin Lakes, NJ, USA) is a surgical haemostatic powder derived from purified plant starch. Its Microporous Polysaccharide Haemospheres are typically absorbed in 24–48 h. It is a thrombin-free, biocompatible and non-pyrogenic agent.
- Surgicel^®^ (Ethicon, 1000 US Highway 202, Raritan, NJ, USA) is a resorbable, sterile haemostatic agent in oxidized and regenerated cellulose (Rayon). Complete absorption occurs in 1/2 weeks.
- TachoSil^®^ (Corza Medical, 247 Station Drive Suite NE1, Westwood, CA, USA) is a sterile patch and a ready-to-use haemostatic agent. It consists of an equine collagen patch coated with human fibrinogen and human thrombin. Complete absorption occurs in 3/4 weeks.
- Hemopatch^®^ (Baxter, Deerfield, IL, USA) is an absorbable collagen pad intended for sealing and haemostasis. Complete absorption occurs in 6/8 weeks.

According to the intraoperatively used haemostats, the whole cohort was divided into 5 groups (Table 2). The number of used haemostats was dependent upon the degree of intraoperative bleeding and at the discretion of the surgeon. The evaluated endpoints included surgical time and haemostatic time, intraoperative blood loss, postoperative chest tube output at 24 h and the total chest tube output, required intra- or postoperative blood products, length of stay, 30-day morbidity and 30-day mortality. Morbidity was analysed and included the need for blood transfusion, cardiovascular shock and ICU stay > 24 h (with hypotension), renal failure (new onset requiring dialysis), sepsis (positive blood cultures and hypotension) and any other postoperative complications.

Haemostatic time was defined as the time between the application of the haemostatic agent and clot formation.

According to a shared protocol, the chest tube was removed as soon as no “air leak” was reported and when the fluid output was lower than 250 mL/day. The patient was usually discharged the day after the chest tube removal. A prolonged “air leak” was considered >5 days.

### 2.2. Statistical Analysis

Data were retrospectively collected in a computerized database and analysed with SPSS software version 11.0.1 for Windows (SPSS Inc., Chicago, IL, USA) under the guidance of a departmental statistician. Continuous data are presented as the mean ± standard deviation or with the median and interquartile range (IQR) depending on the distribution of the data, and we compared through Student’s t test, assuming unequal variances.

Propensity score matching was used to estimate the effect of ARISTA™ AH on various intra- and postoperative parameters (continuous and binary outcome modelling). The propensity scores were estimated using logistic regression based on age, sex, comorbidities, previous cancer, previous lung cancer, tumour location, surgeon expertise, approach, the extent of resection, energy usage and application site. One-to-one nearest-neighbour matching was used. A total of 450 patients from the original 482-patient cohort were matched according to ARISTA™ AH use (yes/no). Good balance was achieved between the ARISTA™ AH and non-ARISTA™ AH group, with all standardized mean differences below 0.2 after matching, all standardized mean differences for squares and two-way interactions between covariates were below 0.15, indicating adequate balance.

To estimate the effect of ARISTA™ AH on outcomes, logistic and linear regression modelling were employed according to the data distribution of the outcome variable with ARISTA™ AH use as the exposure, along with covariates and their interaction as predictors. We included full matching weights in the estimation. The comparisons () function in the marginal effects package was used to perform g-computation in the matched sample to estimate the average treatment effect of the treated population (ATT). Cluster–robust variance was used to estimate its standard error with matching stratum membership as the clustering variable.

All analyses were conducted using R 4.2.3 and the *MatchIt, cobalt, sandwich, lmtest and marginal effects* packages [14,15,16,17,18].

### 2.3. Ethical Statement

This study is a retrospective analysis of standard surgical procedures, conducted in accordance with the Declaration of Helsinki and approved by our internal institutional review board (IRB).

## 3. Results

We included 482 patients without significant difference in preoperative characteristics (age, sex, comorbidities, procedure type) between the groups. All patients’ characteristics are summarized in Table 2.

The majority of procedures were lobectomy (328, 68%), and a robotic assisted procedure was used in 385 patients (80%).

In all 482 patients, haemostasis was achieved using a haemostatic agent: ARISTA™ AH alone was used in 226 cases, SURGICEL in 121, a combination of ARISTA and SURGICEL in 23, a combination of ARISTA and a patch in 4 and a combination of SURGICEL and a patch in 105. In the remaining three patients, the used haemostatic agent was not specified.

All the haemostatic agents are “ready to use”, so there was no time needed to prepare the haemostats. The application of both patches (Tachosil and Haemopatch) lasted 2–3 min, while ARISTA and SURGICEL were applied and left in the blood loss site.

The median surgical time was 100 min (IQR 80–120) with a median haemostatic time of 3 min (IQR 2–8).

The patches (Tachosil and Haemopatch) were applied in 109 cases, when a lung injury occurred, causing an air leak. The haemostatic agents were generally applied to control the bleeding in the lymphadenectomy sites (367 patients; 76%). In 115 patients (24%), the haemostats were used in different sites (e.g., minor bleeding on hilar vessels, oozing from parenchymal stapler line, oozing from pleural adherences).

The majority of patients (467; 87%) had only one chest drain (24 or 28 Ch), while 15 (3%) had two drainages (28 Ch). The median chest tube duration was 3 days (IQR 1–4), and the median hospital stay was 5 days (IQR 4–7).

Postoperative complications were reported in 153 patients (31.7%). No 30- and 90-day mortality occurred, and no allergic reactions to the haemostatic agents were reported.

On postoperative day one (POD#1), the fluid was haemoserous in 98%; in seven cases, it was sanguineous; and in two cases, chyle was noticed.

The median quantity of fluid on the first day was 150 mL (IQR 93–271), and the median total amount of drain fluid was 340 mL (IQR 200–600) (Table 3).

Six patients underwent redo surgery (two for bleeding, one for oncological reasons and three for air leak). No bleeding, blood transfusion or redo surgery was observed in the ARISTA™ AH group.

Eleven covariates were employed for matching as detailed in the methodology. Distribution of propensity scores and covariate balancing are displayed in Supplementary Figures S1 and S2.

### 3.1. Univariate Linear Regression Modelling

We explored the effect of ARISTA™ AH application on the length of stay (days), intraoperative blood loss (mls), duration of surgery (mins), chest drain duration (days) and total drain volume loss (mls) using a linear regression model in a univariate manner. The use of ARISTA™ AH application resulted in a significant reduction in total drain volume by 135 mls (standard error 53.9; *p* = 0.012). The use of ARISTA™ AH did reduce the average length of a hospital stay (−1.47 days) and the duration of chest drainage (−0.596 days), albeit not significantly (Table 4).

### 3.2. Multivariate Regression Modelling with Covariate Interaction

The length of stay, intraoperative blood loss, duration of surgery, chest drain duration and total drain volume loss using a linear regression model in a multivariate manner were not significantly impacted by Arista application (Table 4).

We explored the impact of Arista use on intraoperative complications, postoperative complications, haemoglobin loss or the need for transfusion and the need for a redo operation (Table 5). Use of Arista significantly reduced the odds of postoperative complications, the need for transfusion and redo surgery.

## 4. Discussion

The primary aim of this study was to evaluate the use of ARISTA™ AH as a topical haemostatic agent in minimally invasive thoracic (MIS) procedures and its impact on postoperative morbidity and the length of stay. Intraoperative and postoperative blood loss are associated with poorer outcomes in thoracic surgery and contribute to unnecessary healthcare costs. Furthermore, the use of blood products carries risks such as adverse reactions and transfusion-related injury [19].

Various haemostatic agents have been employed in thoracic surgery, with haemostatic powders like ARISTA™ AH recently introduced. A randomized controlled trial (RCT) of 291 surgical patients across nine centres and three specialties (general, cardiac and orthopaedic surgery) demonstrated the superiority of ARISTA™ AH compared to controls. This study reported that 90.3% of patients treated with ARISTA™ AH achieved complete haemostasis within five minutes (three minutes in cardiac surgery) versus 80.4% in the control group (*p* < 0.0001). Additionally, ARISTA™ AH achieved a shorter time to haemostasis for the first treated lesion (1 min vs. 2 min for control; *p* = 0.002) [20].

Bruckner et al. conducted a retrospective analysis of 240 patients undergoing complex cardiothoracic procedures, comparing 137 patients treated with ARISTA™ AH to 103 control patients. Their findings highlighted improved short-term outcomes in the ARISTA™ AH group, including significantly reduced haemostasis time (93.4 ± 41 min vs. 107.6 ± 56 min; *p* = 0.02), lower chest tube output at 48 h (1594 ± 949 mL vs. 2112 ± 1437 mL; *p* < 0.001) and reduced transfusion requirements (2.4 ± 2.5 units vs. 4.0 ± 5.1 units; *p* ≤ 0.001) [21].

Despite these findings, there is a lack of data on the routine use of ARISTA™ AH following MIS lung resections. In this study, we focused on the impact of ARISTA™ AH on intraoperative time, chest tube output, complications, reoperations and the length of postoperative stay. Our analysis revealed that patients treated with ARISTA™ AH experienced a significant reduction in total drain volume by 135 mL (standard error 53.9; *p* = 0.012). Furthermore, ARISTA™ AH use significantly reduced the odds of postoperative complications, transfusion requirements and reoperation.

Although numerous topical absorbable haemostatic agents are available, limited clinical evidence compares their features—such as ease of use, application method, absorption time and storage requirements—or their effectiveness. Most patients in this study were treated intraoperatively with two similar mechanical haemostatic agents, ARISTA™ AH and SURGICEL™, both used for minor to moderate bleeding control.

ARISTA™ AH has proven to be a safe, effective and fast-acting haemostat, particularly useful in minimally invasive surgery to control minor bleeding following lymphadenectomy, including in challenging areas like beneath the recurrent laryngeal nerve, and for persistent oozing along parenchymal and vascular stapler lines. ARISTA™ AH is easy to apply, ready to use and can be left in situ due to its short absorption time, avoiding foreign body retention and potential misinterpretation on postoperative imaging.

The health economic benefits of ARISTA™ AH are promising, given the reduced incidence of postoperative morbidity, shorter hospital stays and less resource utilization. Zaraca et al. demonstrated similar economic advantages in a randomized trial of sealants to reduce postoperative air leaks, which were associated with decreased morbidity and costs [22]. Future investigations into cost-effectiveness ratios or cost–benefit analyses are warranted to further evaluate these economic impacts.

In conclusion, our findings suggest that the intraoperative use of ARISTA™ AH alone is sufficient for minor to moderate bleeding control. Its use can help reduce costs associated with postoperative bleeding, complications, prolonged hospital stays and surgical time, while also enhancing recovery pathways as part of enhanced recovery after surgery (ERAS) protocols.

## 5. Limitations

The present study has several limitations. The retrospective nature makes it prone to selection bias. Moreover, the group of patients are not homogeneous in terms of the number of treated patients’ subjects.

## 6. Conclusions

While considering the limitations of this study, we believe that a critical evaluation of a large cohort of patients can help to determine the effectiveness of this agent in minimally invasive thoracic procedures.

ARISTA^TM^ AH is a safe and effective haemostat that can help surgeons to control modest to limited bleeding and oozing during surgical procedures with good short-term outcomes for treated patients.

## Figures and Tables

**Table 1:** Summary of haemostatic products.

Group	Category	Active Principle	Example
ACTIVE	Thrombin	Bovine-derived thrombin Human plasma-derived thrombin with porcine gelatine sponge Recombinant thrombin	Thrombin-IMI^®^ Evithrom^®^ Recothrom^®^
	Collagen	Collagen	Avitene™ MCH Helistat^®^ Helitene^®^
COMBINED	Thrombin + mechanical agent	Gelatine plus thrombin	Floseal^®^ Gelfoam^®^ Vitagel^®^
	Fibrin sealant	Human plasma-derived Human pooled plasma and equine collagen	Tisseel^®^ Vistaseal^®^
NON-ACTIVE	Mechanical haemostatic agents	Porcine gelatine Oxidized cellulose Polysaccharide spheres	Surgiflo^®^ Surgifoam^®^ Surgicel^®^ (Tabotamp) ARISTA™ AH

**Table 2:** Patients’ characteristics.

	All	Arista	Surgicel	Arista + Surgicel	Arista + Patch	Surgicel + Patch	*p*-Value
N	482	226	121	23	4	105	
Sex (male)	223 (46.3)	113 (50)	42 (34.7)	14 (60.9)	2 (50)	51 (48.6)	0.041
Age Mean (SD) Median (IQR)	68.9 (10.6) 70 (63–76)	67.1 (10.8) 69 (61–75)	69.9 (9.1) 71 (65–76)	72.7 (7) 73 (67–78)	75.8 (8.4) 76 (69–83)	71 (11.2) 72 (67–79)	0.002
Patients with comorbidity (%)	320 (70.0)	144 (68.6)	115 (95)	15 (65.2)	4 (100.0)	70 (72.2)	0.783
Pulmonary (COPD, IPF)	309 (66.9)	70 (33.8)	110 (90.9)	9 (39.1)	2 (50.0)	95 (90.4)	<0.001
Hypertension	350 (76.3)	105 (51.5)	100 (82.6)	14 (60.9)	3 (75)	60 (57.1)	<0.001
Renal (CKD)	245 (53.0)	11 (5.3)	12 (9.9)	6 (26.1)	0 (0)	15 (14.3)	<0.001
Diabetes	262 (57.2)	30 (14.5)	18 (14.8)	7 (31.8)	0 (0)	10 (9.5)	<0.001
Other	283 (61.3)	50 (24.2)	12 (9.9)	5 (21.7)	0 (0)	9 (8.5)	<0.001
Previous cancer	148 (31.9)	71 (34.0)	38 (31.4)	6 (26.1)	1 (25)	31 (29.8)	0.652
Postoperative complications (%)	153 (31.7)	51 (22.6)	44 (36.4)	7 (30.4)	0 (0)	51 (48.6)	<0.001
Prolonged air leak	45 (9.3)	14 (6.1)	9 (7.4)	4 (17.0)	0 (0)	18 (17.1)	<0.001
Infection/chylothorax	23 (4.7)	6 (2.6)	8 (6.6)	1 (4.3)	0 (0)	8 (7.6)	0.844
Atrial fibrillation	28 (5.8)	9 (4)	7 (5.8)	1 (4.3)	0 (0)	11 (10.5)	0.824
Lung atelectasis/respiratory failure	27 (5.6)	8 (3.5)	9 (7.4)	1 (4.3)	0 (0)	9 (8.5)	0.828
Ileus	22 (4.6)	11 (4.8)	7 (5.8)	0 (0)	0 (0)	4 (3.8)	0.824
Unexpected ICU readmission (%)	8 (1.6)	3 (1.3)	4 (3.3)	0 (0)	0 (0)	1 (0.9)	0.883
Stroke	2 (0.4)	1 (0.4)	1 (0.8)	0 (0)	0 (0)	0 (0)	0.902
Acute renal injury	2 (0.4)	1 (0.4)	1 (0.8)	0 (0)	0 (0)	0 (0)	0.902
Pneumonia and respiratory failure	4 (0.8)	1 (0.4)	2 (1.6)	0 (0)	0 (0)	1 (0.9)	0.913

**Table 3:** Intra- and postoperative variables.

	All	Arista	Surgicel	Arista + Surgicel	Arista + Patch	Surgical + Patch
N	482	226	121	23	4	105
Duration of surgical procedure, min						
Mean (SD)	105.8 (36.0)	103.0 (40.8)	102.2 (25.9)	132.2 (53.7)	96.2 (21.7)	109.9 (28.0)
Median (IQR)	100 (80; 120)	90 (70; 120)	100 (90; 120)	120 (80; 175)	102 (91; 107)	110 (90; 120)
Intraoperative blood loss						
Mean (SD)	44.9 (62.9)	43.3 (61.8)	41.0 (65.0)	101.3 (94.2)	33.8 (31.5)	41.9 (50.1)
Median (IQR)	20 (10; 50)	20 (10; 50)	20 (10; 50)	80 (50; 100)	22 (17; 39)	20 (10; 50)
Hospitalization, Day						
Mean (SD)	6.6 (6.6)	5.4 (3.3)	7.2 (7.1)	6.2 (1.8)	5.2 (2.1)	8.6 (10.2)
Median (IQR)	5 (4; 7)	5 (3; 6)	5 (4; 8)	6 (5; 6)	5 (4; 6)	7 (4; 10)
Chest tube duration, Day						
Mean (SD)	4 (4.7)	3.6 (3.5)	3.9 (4.6)	5 (1.7)	1.5 (1.0)	4.7 (6.9)
Median (IQR)	3 (1;4)	3 (1; 4)	3 (1;4)	5 (4;5)	1 (1;1)	2 (1;4)
Total amount of drain, ml						
Mean (SD)	464.3 (439.6)	355.2 (328.4)	544.9 (604.5)	848.0 (369.4)	183.8 (214.4)	521 (390.4)
Median (IQR)	340 (200; 600)	250 (160; 400)	425 (260; 600)	800 (675; 1000)	82 (72; 194)	445 (232; 702)
Quantity of fluid at first day, mL						
Mean (SD)	200.9 (171.9)	139.8 (140.3)	269.9 (204.3)	321.7 (136.4)	132.5 (112.1)	226.0 (153.4)
Median (IQR)	150 (93; 271)	100 (50; 150)	200 (150; 345)	300 (250; 400)	82 (72; 142)	200 (110; 300)
Patients with comorbidity (%)	320 (70.0)	144 (68.6)	87 (72.5)	15 (65.2)	4 (100.0)	70 (72.2)
Surgeon, Fellow (%)	52 (10.8)	33 (14.6)	10 (8.3)	4 (17.4)	0 (0.0)	5 (4.8)
Prolonged Air leak, (%)	7 (2.2)	3 (4.8)	0 (0.0)	3 (13.0)	- (-)	1 (1.0)
Surgical Procedure						
- Lobectomy (%)	328 (68.0)	152 (67.3)	83 (68.6)	18 (78.3)	2 (50.0)	73 (69.5)
- Segmentectomy (%)	154 (32.0)	74 (32.7)	38 (31.4)	5 (21.7)	2 (50.0)	32 (30.5)
Energy device						
- no	385 (80.7)	168 (74.3)	101 (87.1)	5 (21.7)	4 (100.0)	104 (99)
- ligasure	66 (13.8)	40 (17.7)	13 (11.2)	12 (52.2)	0 (0.0)	1 (1.0)
- harmonic	26 (5.5)	18 (8.0)	2 (1.7)	6 (26.1)	0 (0.0)	0 (0.0)
Quality first day						
- serum-hematic (%)	414 (97.9)	166 (99.4)	117 (96.7)	19 (82.6)	4 (100.0)	105 (100.0)
- hematic (%)	7 (1.7)	1 (0.6)	2 (1.7)	4 (17.4)	0 (0.0)	0 (0.0)
- chylus (%)	2 (0.5)	0 (0.0)	2 (1.7)	0 (0.0)	0 (0.0)	0 (0.0)

**Table 4:** Univariate and multivariate linear regression modelling.

Variable	Estimate	SE	95% CI	*p*-Value
UNIVARIATE				
Length of stay (days)	−1.47	0.806	−3.05–0.108	0.068
Intraoperative blood loss (mls)	9.42	5.99	−2.31–21.2	0.115
Duration of surgery (mins)	1.01	3.44	−5.73–7.76	0.769
Chest drain durations (days)	−0.596	0.462	−1.5–0.31	0.198
Total drain volume (mls)	−135	53.9	−241–29.5	0.012
MULTIVARIATE				
Length of stay (days)	−0.792	0.85	−2.46–0.873	0.351
Intraoperative blood loss (mls)	0.533	9.13	−17.4–18.4	0.953
Duration of surgery (mins)	−0.981	3.52	−7.87–5.91	0.351
Chest drain durations (days)	−0.572	0.24	−1.6–0.455	0.275
Total drain volume (mls)	−126	79.4	−282–29	0.111

**Table 5:** Multivariate logistic regression modelling with covariate interaction.

Variable	Estimate (Odds Ratio)	95% CI	*p*-Value
Intraoperative complications	1	1–1	NS
Postoperative complications	0.383	0.248–0.591	<0.001
Hb loss or transfusion	8.8 × 10^−8^	1.29 × 10^−8^–6.11 × 10^−7^	<0.001
Redo surgery	3.27 × 10^−8^	4.85 × 10^−9^–2.2 × 10^−7^	<0.001

## Data Availability

The data underlying this article will be shared on reasonable request to the corresponding author.

## Supplementary Materials

The following supporting information can be downloaded at: https://www.mdpi.com/article/10.3390/jcm14010085/s1, Figure S1. Distribution of Propensity Scores; Figure S2. Covariate Balance.
