# Supplementary material for: Absorbable Powder Haemostat Use in Minimally Invasive Thoracic Surgery
Source: J Clin Med. 2024 Dec 27;14(1):85. doi: 10.3390/jcm14010085 (PMC11722334; doi:10.3390/jcm14010085)
Supplement: Supplementary file 1 [file jcm-14-00085-s001.zip › jcm-3326823-supplementary.pdf]

Supplementary Figures

Supplementary Figure S1. Distribution of Propensity Scores

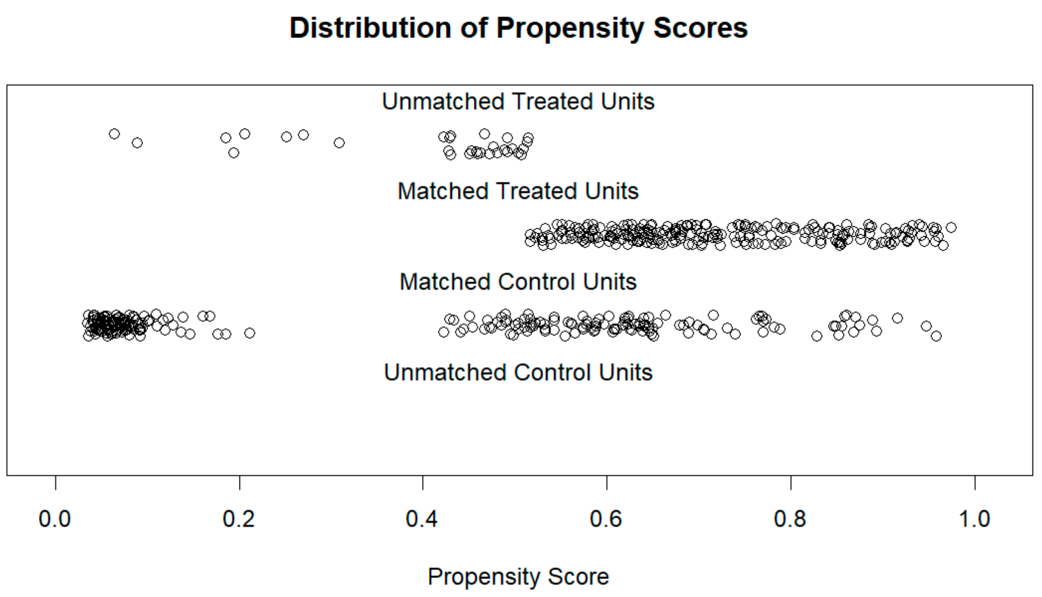

Supplementary Figure S2. Covariate Balance

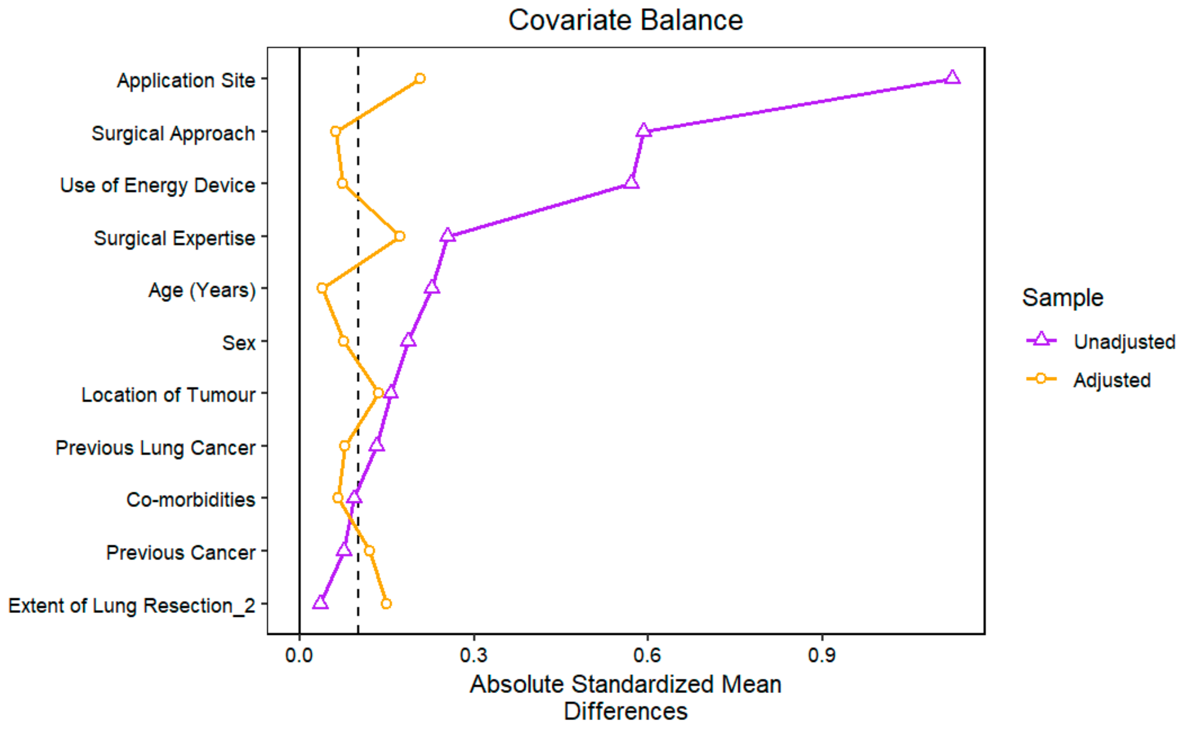
